# Magnetic Beads-Based Electrochemical Label-Free DNA-Bioassay for the Detection of Peanut Allergen Ara h2 in Food Matrices

**DOI:** 10.3390/bios16070387

**Published:** 2026-07-17

**Authors:** Juan Pablo Hervás-Pérez, Sergio Izcara, Marta Sánchez-Paniagua

**Affiliations:** 1 Department of Chemistry in Pharmaceutical Sciences, Faculty of Pharmacy, Complutense University of Madrid, 28040 Madrid, Spain; jphervas@ucm.es; 2 Faculty of Health Sciences—HM Hospitals, Camilo José Cela University, Villanueva de la Cañada, 28692 Madrid, Spain; sergio.izcara@ucjc.edu; 3 HM Hospitals Health Research Institute, 28015 Madrid, Spain

**Keywords:** DNA assay, peanut allergen, label free, sandwich format

## Abstract

The reliable detection of the peanut allergen Ara h2 in processed foods remains a major challenge, since thermal and high-pressure treatments can alter protein structure and limit the performance of immunoassays. DNA-based methods provide a robust alternative to this approach. In this work, a highly sensitive label-free electrochemical genoassay for Ara h2 DNA detection was developed using streptavidin-coated magnetic beads (MBs). A biotinylated capture probe (CP) immobilized on the MBs’ surface enabled specific target recognition through a sandwich hybridization strategy with a secondary probe, allowing for direct electrochemical detection without enzymatic labels. Two transduction strategies were evaluated: (i) electrochemical impedance spectroscopy (EIS) with ferri/ferrocyanide as a redox probe, and (ii) differential pulse voltammetry (DPV) using methylene blue. The ferri/ferrocyanide-based EIS approach showed the best sensitivity and discrimination between hybridized and non-hybridized states. A linear dependence was observed with the concentration of the synthetic Ara h2 target over the 0.05 to 20 nM range, with a detection limit of 0.025 nM. CP-MBs showed good stability for at least 20 days. Applicability was demonstrated in soy beverages, rice beverages, and low-fat cow’s milk, with recoveries close to 100% and negligible matrix effects.

## 1. Introduction

Food allergies affect approximately 8% of children and up to 10% of adults in developed countries, representing a growing public health concern due to their increasing prevalence and the risk of severe immune-mediated reactions [1,2]. Among them, a peanut allergy is considered one of the most clinically significant food allergies, because of its persistence throughout life, very low eliciting dose, and association with severe and potentially fatal allergic reactions [3,4].

Peanut allergenicity is mainly attributed to the Ara h protein family, among which Ara h1 and Ara h2 are the most clinically relevant biomarkers. Although Ara h1 is recognized by more than 90% of peanut-allergic patients, Ara h2 is considered the most potent allergen because of its high IgE-binding affinity, remarkable resistance to digestion, and strong correlation with severe allergic responses [5,6,7]. Consequently, the reliable detection of Ara h-related biomarkers in food products is essential to improve food safety and prevent accidental allergen exposure.

Current analytical methods for peanut detection, including enzyme-linked immunosorbent assays (ELISAs), mass spectrometry, and polymerase chain reaction (PCR), provide high sensitivity and selectivity but generally require expensive instrumentation, labor-intensive procedures, and specialized personnel, limiting their applicability for rapid and decentralized analysis. Electrochemical biosensors have therefore emerged as attractive alternatives because of their low cost, portability, rapid response, and compatibility with point-of-care and on-site testing [8,9].

Most reported electrochemical platforms for peanut allergen detection are based on antibodies or aptamers as biorecognition elements. Although immunosensors provide excellent affinity and specificity, their performance may be compromised by protein denaturation and relatively high production costs. Aptamers offer improved chemical stability but still require complex selection and optimization procedures. Furthermore, food processing treatments such as heating and high-pressure processing can significantly alter protein structure and immunoreactivity, potentially reducing the reliability of protein-based analytical approaches. In contrast, DNA-based strategies detect specific genetic sequences rather than protein epitopes and therefore provide a robust alternative for processed food analysis. Electrochemical genosensors combine the intrinsic selectivity of nucleic acid hybridization with the advantages of electrochemical transduction, enabling highly sensitive and label-free analytical formats [10,11,12].

Despite these advantages, only a limited number of DNA-based biosensors have been reported for peanut allergen detection, and even fewer specifically target Ara h2 [13]. The first electrochemical DNA biosensor reported for Ara h2 detection was based on the formation of binary self-assembled monolayers (SAMs) on screen-printed gold electrodes (SPEAu) [14]. This platform employed a sandwich hybridization format with enzymatic detection, demonstrating high selectivity as a proof of concept, although it was not validated in complex food matrices. More recently, the same SAM formation strategy has been retained, but incorporating gold nanoparticles as a signal amplification element and shifting toward a label-free electrochemical transduction format, which enabled improved analytical performance and successful detection in real food samples [15]. However, surface-confined strategies based on direct probe immobilization onto the electrode generally involve multiple preparation steps and may reduce flexibility in assay design.

Magnetic bead (MBs) technologies are widely used in modern bioanalytical workflows, including nucleic acid extraction, immunoassays, exosome isolation, and molecular diagnostics, owing to their operational simplicity, efficient target capture, rapid magnetic separation, scalability, and compatibility with automated platforms [16,17]. MBs offer key advantages such as high surface-to-volume ratio, efficient functionalization, and facile manipulation under external magnetic fields, enabling rapid separation and washing steps while minimizing matrix interferences. Moreover, by decoupling the biorecognition process from the electrode surface, magnetic platforms provide greater control over hybridization conditions and improve assay reproducibility [18,19]. In this context, the use of MBs as solid support for DNA immobilization represents a compelling alternative to traditional SAM-based approaches that enables a more flexible and simplified workflow, reducing assay complexity while maintaining high analytical performance. However, despite these advantages, no MB-based electrochemical genoassay has yet been reported for Arah2 peanut allergen detection. Recently, Campuzano et al. reported a magnetic bead-based electrochemical platform for peanut DNA detection targeting the chloroplast trnH-psbA marker [20]. Although this approach demonstrated the potential of MB-based electrochemical genosensing for food analysis, it was not directed toward an allergen-related genetic target. To the best of our knowledge, no label-free magnetic bead-based electrochemical genosensor targeting the clinically relevant Ara h2 gene has been reported to date.

In this work, we developed a magnetic bead-based electrochemical label-free DNA biosensor for Ara h2 detection using a sandwich hybridization format. Two electrochemical transduction strategies (electrochemical impedance spectroscopy (EIS) employing ferri/ferrocyanide as an external redox probe and differential pulse voltammetry (DPV) using methylene blue as a DNA redox indicator) were comparatively assessed. The best-performing approach was subsequently optimized and validated for Ara h2 quantification in processed food matrices, providing a sensitive and operationally simple analytical platform for food allergen monitoring.

## 2. Materials and Methods

### 2.1. Apparatus

MBs were employed as the solid support for biosensor development. The modification of the beads and the hybridization and labeling assays were performed in a 12-tube mixing wheel (Dynal MX1) and magnetic separations were carried out with a magnet DynaMag2, both from Life Technologies (Waltham, MA, USA). Unless otherwise stated, all measurements were performed in triplicate using independently prepared MB batches.

Electrochemical measurements were carried out with disposable screen-printed carbon electrodes (SPCEs, DropSens-110, Madrid, Spain), using a PGSTAT101 potentiostat with NOVA 1.9 software (EcoChemie, Utrecht, The Netherlands). Each device comprises three electrodes printed on a ceramic substrate: a carbon working electrode (~4 mm diameter), a Ag pseudo-reference electrode, and a carbon counter electrode. A DropSens^®^ connector was used to interface the screen-printed cells with the potentiostat.

pH measurements were performed with a Crison micropH 2001 pH-meter (Metler Toledo, Madrid, Spain). UV–Vis absorbance measurements were performed using a Genesys 10 spectrophotometer (Thermo Scientific, Madrid, Spain).

### 2.2. Reagents and Solutions

Hexaammineruthenium (III) chloride, hexaammineruthenium (II) chloride, potassium hexacyanoferrate (II), potassium hexacyanoferrate (III), methylene blue, casein and 20× saline sodium phosphate EDTA (SSPE; 0.2 M phosphate buffer, 2.98 M NaCl, 20 mM EDTA, pH 7.4) were purchased from Sigma-Aldrich (Madrid, Spain). Ethanol, potassium chloride, sodium chloride and Tween-20 were obtained from Panreac (Barcelona, Spain). All reagents were of analytical grade and used without further purification. Deionized water was obtained from a Millipore Milli-Q purification system (18.2 MΩ·cm at 25 °C). Streptavidin-coated MBs (Dynabeads MyOne Streptavidin C1, 1 µm diameter; SA-MBs) were purchased from Thermo Fisher Scientific (Spain).

The buffer solutions used in this work were as follows: (i) washing buffer (buffer 1), consisting of 2× SSPE containing 0.01% (*v*/*v*) Tween-20; (ii) immobilization and hybridization buffer (buffer 2), composed of 2 × SSPE with 0.75 M NaCl, pH 7.4; and (iii) measurement buffer (buffer 3), consisting of 0.1 M phosphate buffer containing 0.1 M KCl, pH 7.4.

Commercial beverage samples were purchased from a local supermarket and used as received without further treatment. The samples included a soy-based beverage, a rice-based beverage, and low-fat cow’s milk.

Oligonucleotide sequences were purchased from Sigma-Aldrich (Sigma Life Science) as lyophilized, desalted salts. The DNA target sequence used was selected based on a previously validated 86-base target region from the Ara h2 gene (GenBank accession no. L77197), originally reported for specific peanut allergen detection [14]. The same target sequence was retained here to ensure methodological consistency and direct comparison with earlier electrochemical biosensing platforms.

To further support the selection of this target region, a BLAST + 2.17.0 version search was performed using the NCBI BLASTn tool (BLAST + 2.17.0 version) with the Megablast algorithm against the NCBI nucleotide collection (nt) database. The analysis revealed 100% query coverage and 100% sequence identity with multiple Ara h2/conglutin-7 sequences from different *Arachis hypogaea* cultivars, as well as orthologous sequences from several Arachis species. These results demonstrate that the selected 86 bp region is highly conserved across peanut cultivars and related Arachis species, supporting its selection as a robust target region for Ara h2-based peanut detection.

For selectivity studies, two non-complementary (nC) probes were designed: one fully non-complementary sequence and another containing three mismatches relative to the target. Table 1 provides a summary of the nucleotide sequences of the DNA strands employed. Stock solutions were prepared using Milli-Q water and stored at −20 °C.

A sandwich hybridization assay format was employed using two contiguous single-stranded oligonucleotides complementary to the target sequence: a capture probe (CP) and a secondary probe (SP). This assay configuration offers two main advantages: it enables thermal denaturation of both the target and SP strands prior to homogeneous hybridization, and it allows the use of a shorter CP immobilized on MBs, which can improve hybridization efficiency and assay reproducibility.

Following this design, both CP and SP were selected to be fully complementary to adjacent regions of the target sequence, thereby forming a perfectly dsDNA structure.

For the thermodynamic analysis using the mFold web server [21], thermodynamic predictions were carried out at 25 °C, considering the ionic conditions corresponding to the immobilization/hybridization buffer. The thermodynamic evaluation of the ssDNA CP revealed the presence of four alternative secondary structures with comparable stability, with Gibbs free energy values ranging from −3.66 to −3.36 kcal/mol. These small energy differences suggest the coexistence of multiple conformations in dynamic equilibrium under the experimental conditions. The negative enthalpy (ΔH ranging from −68.0 to −58.2 kcal/mol) and entropy (ΔS ranging from −209.8 to −182.9 cal·K^−1^·mol^−1^) values are consistent with the formation of intramolecular base-pairing interactions, likely involving short and transient stem regions. The predicted melting temperatures (41.5–45 °C) further support the structural stability of the probe, ensuring that both secondary structures remain folded under the experimental working conditions.

In contrast, thermodynamic analysis of the target sequence revealed the formation of a single secondary structure, exhibiting a Gibbs free energy value of −14.22 kcal/mol. The corresponding enthalpy (ΔH = −191.20 kcal/mol) and entropy (ΔS = −593.5 cal·K^−1^·mol^−1^) values are consistent with the formation of stable intramolecular base-pairing interactions. The predicted melting temperature (Tm = 48.9 °C) indicates a moderate structural stability under the experimental conditions. Overall, these results suggest that the target sequence adopts a well-defined folded conformation; however, this structure is not expected to significantly hinder subsequent hybridization with the CP.

Despite the presence of these intramolecular structures in both the CP and the analyte, hybridization analysis of the probe–target duplex demonstrated a highly favorable intermolecular interaction, characterized by a Gibbs free energy change of −55.5 kcal/mol and a predicted melting temperature of 80.8 °C. The large difference between the free energy associated with duplex formation and those corresponding to the individual secondary structures indicates that CP–target hybridization is strongly favored and thermodynamically dominates over intramolecular folding. These data demonstrate the spontaneous and stable hybridization between the target and the CP under experimental conditions.

### 2.3. Procedures

#### 2.3.1. Formation of the Bioconjugated SP-Arah2-CP-SA-MBs

**Figure 1:**
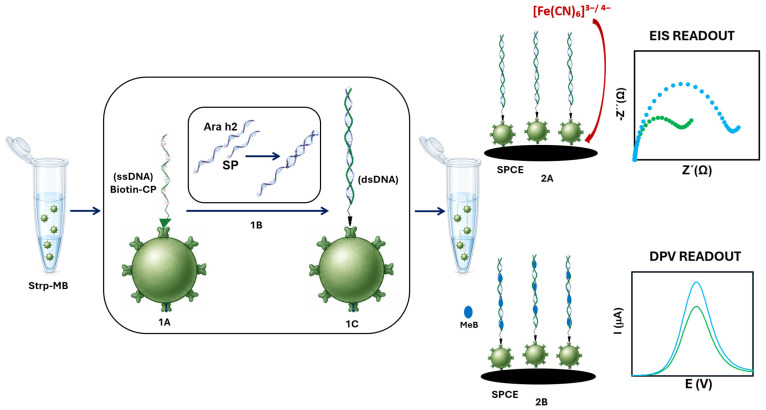
Schematic representation of the bio-magnetoassay for Ara h2 DNA detection. Functionalization of streptavidin-coated MBs with a biotinylated CP (**1A**), homogeneous hybridization (**1B**) and formation of the CP–target–SP DNA sandwich hybrid (**1C**). Label-free electrochemical detection via EIS using the ferri/ferrocyanide redox probe (**2A**) and DPV using MeB as a redox indicator (**2B**).

For the biomodification of the MBs, 5 µL of the commercial SA-MBs (10 mg/mL) was transferred to an Eppendorf tube and diluted with 245 µL of buffer 1. The beads were subjected to two consecutive washes using 250 µL of the same buffer to remove preservatives and ensure clean surfaces. Following washing, the beads were resuspended in 250 µL of a solution containing 1 µM biotinylated CP prepared in buffer 2 (Figure 1(1A)). All immobilization and hybridization steps were carried out in a final reaction volume of 250 µL unless otherwise specified, ensuring consistent bead-to-solution ratios across experiments. The resulting suspension was incubated at room temperature for 30 min under gentle agitation to allow for CP immobilization and then washed twice with 250 µL of buffer 1.

Since a sandwich-type format was employed, two hybridization steps were involved. First, homogeneous hybridization was performed in solutions by mixing the target Ara h2 DNA with 0.5 μM SP in buffer 2 (final volume: 250 µL). The SP acts as a secondary recognition probe and was intentionally used in excess relative to the target DNA in each experiment. Although the absolute SP concentration varied during the study, it was always maintained at a molar excess over the target DNA. This strategy ensures efficient hybridization of the target molecules in solution and maximizes the formation of the CP–target–SP sandwich complex over the entire range of target concentrations evaluated.

The mixture was heated at 98 °C for 5 min and immediately cooled in an ice bath for 5 min (Figure 1(1B)). These conditions were selected based on preliminary optimization experiments showing maximal disruption of secondary structures and efficient re-annealing of the SP–target duplex. The mixture was left 15 min at room temperature to facilitate hybridization. Subsequently, heterogeneous hybridization took place by incubating pre-hybridized complexes with CP-MBs. With this aim, 250 µL of the homogenous hybridization mixture was used to resuspend the washed microparticles. The heterogeneous hybridization reaction proceeded for 45 min at room temperature under rotation (Figure 1(1C)).

After hybridization, MBs were magnetically separated and washed repeatedly with buffer 1 to remove non-specifically bound sequences, thereby minimizing background interference.

At each stage—CP immobilization, target hybridization, and pre-measurement preparation—the MBs were repeatedly washed with buffer and collected using a magnetic separator. This approach allowed for efficient pre-concentration of the target and removal of potential interfering species, ensuring high sensitivity and specificity in the label-free electrochemical assay. Washing efficiency was confirmed in preliminary experiments by monitoring the absorbance at 260 nm of the supernatant, as a qualitative indicator of unbound oligonucleotide removal.

#### 2.3.2. Label-Free Electrochemical Detection

The label-free electrochemical detection of Ara h2 DNA was performed using two complementary transduction strategies: (i) an external redox probe-based impedance approach (Figure 1(2A)) and (ii) an interfacial redox indicator-based voltammetric approach using MeB (Figure 1(2B)). Both methods were applied to MBs-bound DNA hybrids captured via the CP–target–SP sandwich format.

For label-free impedance-based detection using an external redox probe, 15 µL of the modified MBs suspension were magnetically confined onto the working electrode surface of a SPCE placed inside a custom-made PMMA holder. The supernatant was then removed, and 50 µL of an external redox probe solution containing either [Fe(CN)_6_]^3−^/[Fe(CN)_6_]^4−^ or [Ru(NH_3_)_6_]^3+^/[Ru(NH_3_)_6_]^2+^ was added onto the electrode surface, ensuring complete coverage of the three-electrode system.

Electrochemical impedance spectroscopy (EIS) was then performed in the presence of the redox probe to monitor changes in charge-transfer resistance (R_ct_) associated with the formation of the DNA sandwich complex.

For voltammetric detection using a redox indicator, the DNA-functionalized 15 µL of the functionalized MBs suspension was assembled on the working electrode surface (inside the PMMA holder) and were immersed in a stirred aqueous solution containing 20 µM of MeB. The system was incubated for 10 min under gentle stirring to allow the binding of MeB to surface-confined ssDNA/dsDNA. The beads were then magnetically retained and a washing step with buffer 3 was performed to remove non-specifically bound dye. Electrochemical measurements were subsequently carried out in fresh buffer solution by differential pulse voltammetry (DPV). The cathodic peak current corresponding to the reduction in MeB at −0.15 V was used as the analytical signal.

### 2.4. Electrochemical Measurements

Before each measurement, the SPCEs were rinsed with ethanol and deionized water, dried under a nitrogen stream, and used without any electrochemical pretreatment. All experiments were performed at room temperature using a new disposable SPCE for each measurement.

A custom-designed holder incorporating a permanent magnet was employed to ensure the stable retention of magnetic beads on the electrode surface during the electrochemical measurements. The SPCE was positioned within the holder with the magnet aligned directly beneath the working electrode. Following deposition of the magnetic beads (MBs) onto the electrode surface, the magnetic field ensured stable immobilization of the particles throughout the electrochemical measurements, providing reproducible localization of the magnetic phase at the electrode interface.

Cyclic voltammetry (CV) and EIS were employed to characterize the interfacial properties of the modified electrode surface during the stepwise fabrication of the genosensor. Quantitative detection was subsequently performed by either EIS or DPV, depending on the redox system employed.

CV experiments were performed in a solution containing 1 mM ferricyanide/ferrocyanide prepared in buffer 3, within a potential window of −0.10 to +0.60 V at a scan rate of 50 mV s^−1^.

EIS measurements were performed in the same redox solution at the open-circuit potential (OCP), applying a 5 mV (peak-to-peak) sinusoidal perturbation over a frequency range from 10^6^ to 0.1 Hz. The impedance spectra were fitted to a Randles equivalent circuit. The Nyquist plots exhibited a high-frequency semicircle corresponding to electron-transfer kinetics, followed by a low-frequency linear region associated with diffusion-controlled processes. The diameter of the semicircle was used to determine R_ct_, which increased progressively during sensor assembly, confirming successful stepwise modification of the electrode surface.

DPV was employed for the quantitative detection of MeB-based interfacial signals. Measurements were performed using the following parameters: pulse amplitude 0.001 V, pulse width 0.05 s, pulse period 0.2 s, and a potential window from +0.3 V to −0.5 V (vs. Ag/AgCl). The cathodic peak current corresponding to the reduction in MeB was used as the analytical signal.

## 3. Results and Discussion

### 3.1. Electrochemical Characterization of the Biosensor Fabrication Steps

The electrochemical behavior of the biosensing platform during the different fabrication and hybridization steps was investigated by EIS using [Fe(CN)_6_]^3−^/^4−^ as the redox probe.

**Figure 2:**
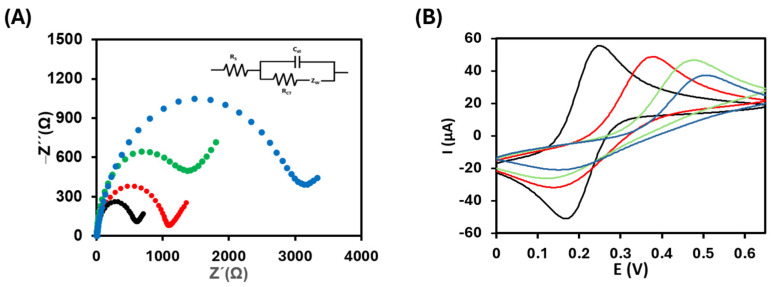
(**A**) EIS spectra and (**B**) CV curves of SPCE (black line), MBs-SPCE (red line), CP-MBs-SPCE (green line) and SP-Arah2-CP—MBs SPCE (1 nM Ara h2) (blue line) in 1 mM [Fe(CN)_6_]^3−/4−^ (buffer 3). The inlet of figure (**A**) is the equivalent circuit used to model impedance data.

Figure 2A shows the Nyquist plots corresponding to the sequential construction of the biosensing architecture. The bare SPCE exhibited a relatively low R_ct_ (620 Ω ± 45 Ω, *n* = 3), indicating fast electron-transfer kinetics between the redox probe and the electrode surface. After MB deposition onto the SPCE surface, an increase in R_ct_ was observed (1100 ± 70 Ω, *n* = 3), attributed to the partial blocking effect generated by the MB layer, which hinders diffusion of the redox species toward the electrode surface. The deposition of CP-MBs on SPCE produced a further elevation in R_ct_ of 1730 Ω (1730 ± 85 Ω, *n* = 3). This higher impedance is associated with the negatively charged phosphate backbone of the immobilized ssDNA, which electrostatically repels the anionic [Fe(CN)_6_]^3−^/^4−^ redox probe and further restricts electron transfer. The largest R_ct_ value was observed after deposition of the SP-Arah2-CP-MBs complexes onto the electrode surface (1 nM Ara h2), reaching 3425 Ω ± 70 Ω (*n* = 3), consistent with the formation of the dsDNA sandwich structure. All impedance spectra were fitted using the equivalent Randles circuit, and the reported R_ct_ values were obtained from the fitted curves. Since the fits were almost superimposable with the experimental data, they were omitted from the figure for clarity.

Overall, the progressive increase in R_ct_ throughout the assembly process demonstrates the successful construction of the sensing platform. These results indicate that the ferri/ferrocyanide redox couple effectively monitors the interfacial changes associated with DNA immobilization and hybridization, enabling the conversion of the biorecognition event into a measurable electrochemical signal.

As preliminary studies, two control experiments were performed. In the first control, MBs were incubated with the hybrid Ara h2-SP in the absence of CP (SP–Ara h2–MBs). In this case, the obtained R_ct_ value (1250 Ω) was slightly higher than that observed for MBs/SPCE alone (1100 Ω), suggesting the presence of a limited degree of nonspecific adsorption of the target or SP onto the MB surface. However, this increase was considerably lower than that observed after specific hybridization (3425 Ω), indicating that nonspecific interactions contribute only marginally to the overall electrochemical response.

In the second control experiment, CP immobilization and SP addition were performed on the MBs in the absence of a target (SP–CP-MBs), and the modified MBs were deposited onto the SPCE surface. The resulting impedance response (1980 Ω) was comparable to that obtained for CP-modified MBs alone (1730 Ω), indicating that the presence of SP does not lead to significant nonspecific adsorption onto the CP-functionalized surface under the selected experimental conditions. This behavior is likely driven by electrostatic repulsion between negatively charged phosphate backbones.

CV measurements were performed to further corroborate the interfacial changes observed by EIS during the stepwise fabrication of the biosensor (Figure 2B). The bare SPCE exhibited a well-defined and quasi-reversible redox behavior, with a peak current (Ip) of approximately 63 µA and a small peak-to-peak separation (ΔEp ≈ 0.08 V), together with an oxidation-to-reduction current ratio (Ip/Ia) of 1.02. These features indicate fast electron-transfer kinetics at the unmodified electrode surface. After the deposition of MBs onto the electrode surface, a decrease in peak current to approximately 45 µA was observed, accompanied by an increase in ΔEp to 0.21 V. This behavior reflects partial blocking of the electrode surface by the insulating bead layer, which slows down electron transfer and reduces the electroactive area available to the redox probe. A decreased in the Ip (40 µA) with a ΔEp to 0.29 V and Ip/Ia ratio 0.55 was obtained after the addition of CP-MBs onto SCPE. These changes are consistent with the formation of a negatively charged DNA layer on the electrode surface, which introduces additional electrostatic repulsion toward the anionic [Fe(CN)_6_]^3−^/^4−^ species and further hinders charge transfer at the interface. The lower peak current (29 µA) was obtained with the modification of SCPE with SP-Arah2-CP-MBs (ΔEp = 0.32 V). This pronounced increase in peak separation indicates a significant reduction in electron-transfer reversibility, in agreement with the formation of the CP–target–SP duplex structure.

### 3.2. Evaluation of Label-Free Electrochemical Detection Strategies

With the aim of achieving a highly sensitive bioassay, two label-free electrochemical transduction strategies were evaluated for the detection of Ara h2 DNA hybridization on MBs: (i) EIS using external redox probes, and (ii) DPV using MeB as an interfacial redox indicator. The two approaches rely on fundamentally different transduction mechanisms: EIS monitors changes in interfacial charge-transfer resistance induced by DNA hybridization, whereas MeB-based DPV detects variations in the interfacial accumulation of a redox-active dye that interacts with DNA through electrostatic and partial intercalation mechanisms. The experiments were carried out under the following initial conditions: 0.5 µM CP for 30 min, 1 µM SP for 25 min, 1 nM Ara h2 target DNA, 25 min homogeneous hybridization, and 30 min heterogeneous hybridization.

The first EIS-based approach was evaluated using two different redox probes: [Fe(CN)_6_]^3−^/^4−^ and [Ru(NH_3_)_6_]^3+^/^2+^.

The Nyquist plots obtained with [Fe(CN)_6_]^3−^/^4−^ exhibited well-defined semicircles, allowing for reliable determination of the R_ct_. In contrast, measurements performed with the ruthenium complex showed poorly defined semicircles and limited variation between ssDNA- and dsDNA-modified MBs, hindering discrimination of hybridization events.

**Figure 3:**
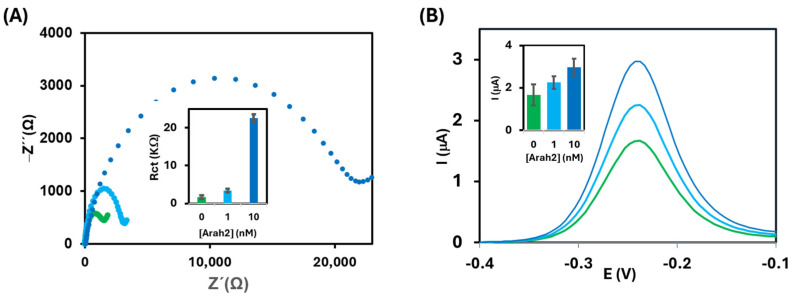
Label-free electrochemical detection of Ara h2 DNA hybridization on MBs. (**A**) EIS response using a ferro/ferricyanide system and (**B**) DPV response using MeB. CP-MBs (green color); 1 nM Ara h2 target DNA (light blue); 10 nM Ara h2 target DNA (dark blue). Insets show the corresponding analytical signals. Error bars represent SD (*n* = 3).

For the ferri/ferrocyanide system, a progressive increase in R_ct_ was observed after immobilization of the CP onto MBs and after hybridization with the Ara h2-SP hybrid, indicating successful formation of the DNA sandwich structure. This response is attributed to increased electrostatic repulsion between the negatively charged [Fe(CN)_6_]^3−^/^4−^ redox probe and the DNA phosphate backbone, together with enhanced steric hindrance upon dsDNA formation, which collectively hinder electron transfer at the electrode/electrolyte interface. Accordingly, dsDNA-modified MBs exhibited significantly higher impedance values than their ssDNA-modified counterparts. Signal-to-background (S/B) ratios of 1.9 and 13.5 were obtained for Ara h2 concentrations of 1 and 10 nM, respectively (Figure 3A).

Conversely, the electrochemical behavior observed with [Ru(NH_3_)_6_]^3+^/^2+^ differed markedly from that obtained with ferri/ferrocyanide. This positively biased redox probe is electrostatically attracted to the negatively charged phosphate backbone of DNA, leading to accumulation of the ruthenium complex within the DNA layer. As a result, electron transfer is facilitated rather than hindered, yielding only small differences in R_ct_ between ssDNA and dsDNA configurations. This reduced discrimination is attributed to the strong electrostatic attraction of the cationic complex to both ssDNA- and dsDNA-modified surfaces, which limits the contrast between hybridized and non-hybridized states.

The second label-free strategy investigated was based on MeB as an electroactive DNA-interacting indicator monitored by DPV. An increase in the cathodic reduction current is observed in dsDNA-MBs-SPCE compared to ssDNA (Figure 3B). MeB interacts with DNA through multiple mechanisms, including electrostatic interactions with the phosphate backbone, preferential binding to guanine bases in ssDNA, and intercalation within dsDNA. The relative contribution of these interactions depends on the DNA conformation and surface density. The resulting signal enhancement after hybridization was moderate, with S/B ratios of 1.3 and 1.8 for Ara h2 concentrations of 1 and 10 nM, respectively. A relatively high background signal was observed for the ssDNA state that can be attributed to MeB interaction with exposed guanine bases in the immobilized CP. After hybridization, the formation of the dsDNA structure promotes additional MeB accumulation through intercalation and electrostatic interactions, leading to an increase in the reduction signal. However, the high initial background limits overall sensitivity.

Overall, among the evaluated label-free approaches, ferri/ferrocyanide-based EIS provided the highest discrimination between hybridized and non-hybridized states, showing an approximately 7.5-fold increase in signal-to-background ratio compared with the DPV-based MB assay, and was selected for subsequent optimization and analytical characterization.

Two measurement strategies were also evaluated after depositing the MBs suspension onto the electrode surface: (i) removal of the supernatant prior to addition of the redox probe and (ii) measurement without removing the supernatant. The first strategy resulted in improved reproducibility, with a coefficient of variation (CV) of 8%, compared to 18% for the second approach. This improvement can be attributed to better confinement of the MB layer at the electrode surface and more controlled interaction between the redox probe and the interfacial biolayer, minimizing diffusion-related variability. Since the ferri/ferrocyanide-based EIS strategy exhibited significantly higher sensitivity, better signal-to-background ratios, and superior analytical performance than the methylene blue approach, only the EIS platform was selected for further optimization. Consequently, additional optimization of methylene blue concentration and incubation time was not pursued.

### 3.3. Optimization of the Experimental Variables

The control studies demonstrate that the pronounced increase in R_ct_ observed after hybridization is predominantly associated with the specific formation of the CP–Ara h2–SP complex, while nonspecific interactions remain relatively low. Nevertheless, the slight increase observed in the control experiments indicates that a minor degree of nonspecific adsorption may still occur. Therefore, additional studies focused on the optimization of experimental variables, including probe concentrations, washing procedures, and incubation times, aiming to further minimize nonspecific interactions and enhance target signal. These studies were performed with a fixed target concentration of 1 nM.

**Figure 4:**
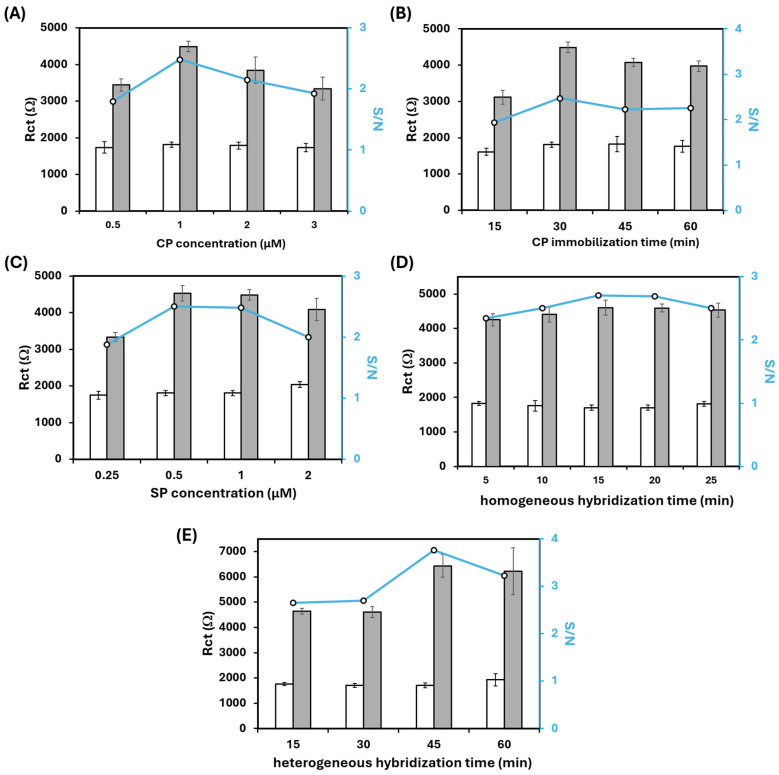
Optimization of the experimental variables for Ara h2 detection, measurements by the impedimetric responses in the absence (white bars) and in the presence (grey bars) of 1 nM Ara h2 target. The resulting S/N values are shown as blue line. Error bars represent the standard deviation (*n* = 3). CP concentration (**A**), CP incubation time (**B**), SP concentration (**C**), homogeneous hybridiation time (**D**), and heterogeneous hybridization time (**E**) were evaluated.

CP concentration was optimized in the 0.5–3 µM range (Figure 4A). The analytical response increased from approximately 3400 Ω at 0.5 µM to 4500 Ω at 1 µM, while the S/N ratio reached its maximum value (~2.5) at this concentration. Higher CP concentrations did not improve the response and led to a slight decrease in the S/N ratio, probably due to steric hindrance effects caused by excessive surface coverage. Therefore, 1 µM CP was selected for further experiments.

The incubation time of the CP onto the MBs was evaluated between 15 and 60 min (Figure 4B). The signal increased significantly up to 30 min, reaching an R_ct_ value close to 4550 Ω, whereas longer incubation times produced negligible changes in both R_ct_ and S/N ratio, indicating saturation of the available streptavidin binding sites.

The SP concentration was optimized in the 0.25–2 µM range (Figure 4C). The maximum analytical response and S/N ratio were obtained at 0.5 µM SP. Lower signal values were observed at 0.25 µM, likely due to insufficient probe availability for efficient sandwich hybridization. At higher concentrations, no further signal enhancement was achieved, while a slight decrease was observed at 2 µM, possibly reflecting less efficient hybridization under probe excess conditions. Therefore, 0.5 µM was selected as the optimal SP concentration for subsequent experiments.

The homogeneous hybridization time between the target DNA and the SP was evaluated from 5 to 25 min (Figure 4D). The response increased progressively up to 15 min, where the highest S/N ratio (~2.8) was achieved. Longer incubation times did not improve the analytical signal, indicating efficient hybrid formation within this period.

Finally, the heterogeneous hybridization time between the preformed hybrid and the CP-modified MBs was optimized between 15 and 60 min (Figure 4E). The maximum response was obtained after 45 min of incubation, reaching an R_ct_ value above 6000 Ω and an S/N ratio close to 4. No significant improvement was observed at 60 min, so 45 min was selected as the optimal heterogeneous hybridization time.

Table 2 provides an integrated overview of the experimental variables considered during the development of the Ara h2 DNA biosensor, describing the range studies performed and the conditions ultimately selected.

Different washing conditions were evaluated to reduce nonspecific adsorption during the biosensor assembly process. Based on previous reports for magnetic bead-based electrochemical genosensors and immunosensors [22,23], washing buffers containing Tween 20 and/or casein were investigated. A modified washing buffer (buffer 1) containing 0.1% (*w*/*v*) casein and 0.01% (*v*/*v*) Tween 20 was evaluated during the washing steps. The incorporation of casein was intended to reduce nonspecific surface interactions through its blocking effect, while Tween 20 acted as a mild surfactant to improve washing efficiency. Although the addition of casein reduced the blank signal, it also caused a significant decrease in the analytical signal obtained in the presence of the target analyte, resulting in a lower signal-to-background (S/B) ratio. These results suggest that excessive surface blocking may partially hinder probe accessibility and negatively affect hybridization efficiency on the MBs surface. Therefore, the washing buffer containing only 0.01% Tween 20 was selected as the optimal condition.

### 3.4. Analytical Characteristics of the Genosensor

**Figure 5:**
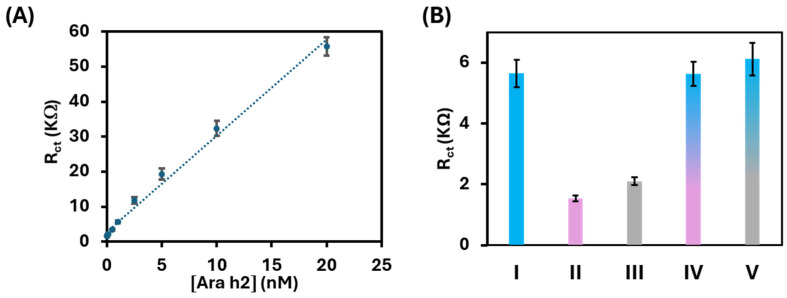
(**A**) Calibration plot and (**B**) interference study. Error bars represent the standard deviation (*n* = 3). In panel (**B**): I, Ara h2; II, nC; III, nC-3; IV, Ara h2 + nC; V, Ara h2 + nC-3.

The analytical performance of the developed bioassay was evaluated by EIS using increasing concentrations of the target Ara h2 DNA sequence. Figure 5A shows the corresponding calibration plot obtained from the variation in R_ct_ as a function of target concentration. Under optimized experimental conditions, the genosensor exhibited a linear response over the concentration range from 0.05 to 20 nM, following the regression equation: R_ct_ (Ω)= (2744 ± 163) C (nM) + (1650 ± 77) (R^2^ = 0.9991). Each calibration point corresponds to the mean value obtained from three independent measurements (*n* = 3) performed at Ara h2 target DNA concentrations of 0.05, 0.10, 0.5, 1, 2.5, 5.0, 10.0, and 20.0 nM. Error bars represent the corresponding standard deviations. The detection (LOD) and quantification (LOQ) limits were calculated according to the conventional criteria of $\bar{x}$B + 3sB and $\bar{x}$B + 10sB, N = 10, respectively, where xB represents the mean blank response and sB corresponds to the standard deviation of ten independent blank measurements. Based on these calculations, the LOD and LOQ were estimated to be 0.025 nM and 0.08 nM, respectively, confirming the high sensitivity of the proposed genosensor toward Ara h2 DNA detection.

The precision of the developed genoassay was evaluated at three Ara h2 target DNA concentrations within the linear working range, corresponding to low (0.05 nM), medium (1 nM), and high (20 nM) concentration levels. Intra-day precision was assessed from ten consecutive measurements performed on the same day using the same biosensor. Inter-day precision was evaluated over five different days using the same device. Reproducibility was investigated using five independently fabricated biosensors, each analyzed in triplicate. As summarized in Table 3, the relative standard deviation (RSD) values were below 8% for all tested concentrations, demonstrating the good precision, reproducibility, and operational reliability of the proposed genosensing platform.

### 3.5. Selectivity and Stability

The selectivity of the developed genosensor was evaluated by comparing the response obtained for 1 nM fully complementary target DNA with those measured in the presence of a non-complementary soybean-derived DNA sequence (nC), previously reported as a target for soybean detection [24], and a three-base mismatched (nC-3) sequence (Figure 5B). The non-complementary sequence produced a signal comparable to the blank, indicating negligible non-specific hybridization. In contrast, the nC-3 sequence generated only ~12% of the signal observed for the fully complementary target, demonstrating strong discrimination against mismatches. When mixtures containing the target together with either nC or nC-3 sequences were tested, the response remained essentially unchanged compared to the target alone, further confirming robust selectivity.

The storage stability of the prepared CP-MBs was evaluated by storing the functionalized MBs at 4 °C in Eppendorf tubes containing PBS buffer, either in the absence or presence of 0.01% Tween-20. At predetermined time intervals, aliquots of the stored CP-MBs were collected and employed to perform the complete hybridization assay followed by EIS measurements. The analytical performance was assessed by monitoring the signal-to-background (S/N) ratio obtained for the target and blank samples. The results demonstrated that the biosensor maintained stable analytical performance for up to 12 days when stored in PBS alone and up to 20 days in PBS supplemented with 0.01% Tween-20. After these periods, a gradual decrease in the S/N ratio was observed. The improved stability in the presence of Tween-20 may be attributed to reduced bead aggregation and enhanced colloidal stability during storage. The obtained stability is comparable to that reported for other DNA [25] or antibody based-biosensors [26] based on MBs. These findings indicate that the functionalized CP-MBs can be prepared in advance and stored under the conditions described prior to biosensor fabrication and analysis.

### 3.6. Application of Real Samples

The accuracy of the method and its indirect assessment of matrix effects were evaluated using spiked commercial soy-based beverage, rice-based beverage, and low-fat cow’s milk (100-fold dilution) samples. As an additional study, the slopes of calibration curves obtained with standard solutions prepared in buffer were compared with those obtained in the three food matrices. Slope values of 2744 ± 163 (buffer), 2799 ± 145 (soy-based beverage), 2865 ± 184 (rice-based beverage), and 2956 ± 201 (low-fat cow’s milk) were obtained. Student’s *t*-test at a significance level of 0.05 and five degrees of freedom was applied to evaluate differences between slopes, yielding t_exp values of 1.350, 1.465, and 1.575 for the soy-based beverage, rice-based beverage, and low-fat cow’s milk, respectively, all below t_tab = 2.571, indicating no significant differences and confirming negligible matrix effects under the studied conditions.

For the recovery study, samples were spiked with different concentrations of peanut Ara h2 DNA and analyzed in triplicate following the EIS protocol (Table 4). Recovery values ranged from 94% to 106%, demonstrating good accuracy and reliability and confirming the suitability of the method for real sample analysis.

### 3.7. Comparison with Other Analytical Methods

A wide range of analytical techniques, including enzyme-linked immunosorbent assays (ELISAs), polymerase chain reaction (PCR), and mass spectrometry, have been employed for peanut detection because of their high sensitivity and selectivity [27,28,29]. However, their routine application is often limited by the need for expensive instrumentation, labor-intensive procedures, and specialized personnel. Electrochemical biosensors have therefore emerged as attractive alternatives owing to their portability, rapid response, low cost, and suitability for decentralized food analysis [30,31].

Among electrochemical platforms, most reported peanut biosensors are based on antibodies or aptamers as recognition elements [32,33]. Although these approaches provide excellent analytical performance, protein instability, batch-to-batch variability, and the structural modifications induced by food processing may compromise the reliability of protein-targeted assays. DNA-based genosensors overcome many of these limitations by targeting stable genetic sequences, offering high sequence specificity, improved chemical stability, and simplified label-free assay formats.

Despite recent advances in biosensing technologies, only a limited number of DNA-based biosensors have been reported for peanut allergen-related DNA detection [13]. Most of the existing genosensors rely on surface immobilization strategies based on self-assembled monolayers (SAMs) for the anchoring of DNA probes onto electrode surfaces. A comparative overview of the main electrochemical DNA-based sensors reported for peanut allergen determination, including their analytical performance and practical characteristics, is presented in Table 5.

The first electrochemical DNA biosensor reported for Ara h2 detection was based on binary SAM formation on screen-printed gold electrodes (SPEAu), coupled with a sandwich hybridization format and enzymatic signal amplification [14]. Although this approach demonstrated high selectivity as proof-of-concept, it required multiple labeling steps and showed limited applicability in complex food matrices. More recently, a label-free electrochemical genosensor based on SAM-modified electrodes and gold nanoparticles has been developed, enabling direct electrochemical transduction and eliminating the need for enzymatic labeling [15].

In this context, the use of MBs as a solid support for DNA immobilization represents a compelling alternative to traditional SAM-based approaches. Magnetic bead-based assays offer a high surface-to-volume ratio, efficient functionalization, and straightforward handling under external magnetic fields, enabling rapid separation and washing steps while reducing matrix interferences [19]. Moreover, by decoupling the biorecognition event from the electrode surface, magnetic platforms allow for improved control over hybridization conditions and enhanced assay reproducibility [34]. As summarized in Table 5, magnetic bead-based platforms also compare favorably with conventional SAM-based genosensors in terms of stability and applicability to complex food matrices, and storage stability, while maintaining competitive analytical performance in terms of linear range and limit of detection.

Recent advances in peanut DNA biosensing have also demonstrated the potential of magnetic bead-based platforms. Campuzano et al. developed a disposable amperometric biotool for peanut detection in processed foods based on streptavidin-coated MBs and enzymatic signal amplification, targeting the chloroplast DNA marker trnH-psbA [20]. While this approach enabled sensitive detection of peanut-derived genetic material, the selected target is a taxonomic marker that confirms the presence of peanuts but does not provide information on specific allergenic proteins responsible for IgE-mediated allergic reactions. In contrast, the present work employs an MB-based label-free genosensing strategy directed toward the Ara h2 gene, which encodes one of the major peanut allergens and is widely recognized as one of the most clinically relevant markers for peanut allergy diagnosis due to its strong association with severe allergic reactions. By targeting an allergen-coding gene rather than a general plant marker, the proposed platform offers greater relevance for allergen-specific food analysis while maintaining the advantages of a simplified label-free assay format.

In addition, the proposed biosensor exhibited an operational stability of approximately 20 days. This performance represents a significant improvement over previously reported label-free Ara h2 genosensors based on SAM-functionalized gold electrodes, which typically retain satisfactory analytical performance for about 10 days under comparable storage conditions [15]. This enhanced stability may be attributed to the strong streptavidin–biotin interaction used for probe immobilization and to the use of MBs as a three-dimensional support, which can mitigate some of the limitations associated with the long-term stability of self-assembled monolayers on electrode surfaces.

**Table 5:** Comparative analysis of electrochemical biosensors for peanut-specific DNA detection.

Peanut Allergen	Transducer	Immobilization Method	Electrochemical Method/Amplification	Linear Range (LOD)	Stability	Real Sample	Ref.
Ara h1	Gold electrode	SAM CP-MCH	EIS/No	10^−6^ to 0.1 nM (0.35 fM)	21 days (87%)	Peanut milk Beverage	[35]
Ara h1	GCE	SAM CP-MCH on graphene–gold nanocomposite	DPV/No	10^−7^ to 10^−4^ nM (0.041 fM)	21 days (82%)	Peanut milk beverage	[36]
Ara h1	GCE	SAM CP-MCH on chitosan–MWCNT composite and spongy gold film.	Chronoamperometry/HRP	3.91 × 10^−10^–1.25 × 10^−6^ nM (1.3 × 10^−8^ nM)	10 days (80.62%)	Real peanut	[37]
Ara h2	SPEAu	SAM-MCH	DPV/Alkaline phosphatase	0.05–50 nM (10 pM)	Not reported	-	[14]
Ara h2	AuNPs-modified SPCE	SAM CP-DTT	SWV/No	0.025–10 nM (0.02 nM)	10 days 15 days (85%) 20 days (65%)	soy-based beverages and cow’s milk	[15]
Peanut DNA (trnH-psbA)	SPCE	magnetic microbeads	Amperometry/HRP	3 × 10^−3^ nM (3 pM)	Not reported	cereal, chocolates, cookies, pesto sauce, muesli and vegetal burger	[20]
Ara h2	SPCE	Magnetic microbeads	EIS/No	0.05 to 20 nM (0.025 nM)	20 (92%)	Soy-based beverage, Rice-based beverage Low-fat cow’s milk	This work

The proposed platform was designed as a disposable assay using screen-printed carbon electrodes and functionalized magnetic beads; therefore, sensor regeneration was not investigated. Although the proposed biosensor showed satisfactory analytical performance in spiked soy-based beverage, rice-based beverage, and low-fat milk samples, the complexity of real food matrices remains an important challenge for practical implementation. Food samples contain a wide variety of endogenous compounds, including proteins, lipids, polysaccharides, and other high-molecular-weight components, which may interfere with DNA accessibility, hybridization efficiency, or electrochemical signal generation. Consequently, the analytical performance observed in spiked samples may differ from that obtained with naturally contaminated food products.

Therefore, additional validation using a broader variety of food matrices and more representative sample types will be necessary to fully assess the robustness, reliability, and applicability of the proposed genosensing platform under real analytical conditions. Such studies would further support its implementation as a routine tool for food allergen monitoring.

Finally, it is important to note that the present work is based on spiked samples containing target DNA sequences. Therefore, the analysis of real food products containing endogenous allergen-related DNA would require prior DNA extraction and purification steps. Future studies will focus on integrating suitable DNA extraction protocols and evaluating the performance of the proposed biosensor using genomic DNA extracted from naturally contaminated or commercial food samples. In addition, the influence of matrix-derived inhibitors on analytical performance will also be systematically investigated. These studies will further support the practical implementation of the proposed platform for routine food allergen monitoring.

## 4. Conclusions

In this work, a novel label-free electrochemical genosensor based on streptavidin-coated magnetic beads (MBs) was successfully developed for the sensitive and selective detection of the peanut allergen Ara h2 DNA sequence in processed food products. The proposed platform combines the advantages of MB-assisted target preconcentration with a sandwich-type hybridization strategy involving a biotinylated capture probe and a secondary probe, enabling efficient and specific recognition of the target sequence. Importantly, the methodology allows direct electrochemical detection without the need for enzymatic labels, simplifying the assay design and reducing operational costs and assay complexity.

Two different label-free electrochemical transduction strategies were evaluated and compared: EIS using ferri/ferrocyanide as an external redox probe, and DPV using MeB as an intercalating redox indicator. Among them, the ferri/ferrocyanide-based EIS approach exhibited superior analytical performance, providing higher sensitivity and better discrimination between hybridized and non-hybridized states.

Under optimized experimental conditions, the developed biosensor demonstrated excellent analytical features, including a low detection limit of 0.025 nM, high selectivity against non-complementary and mismatched DNA sequences, and good reproducibility with coefficient of variation values below 8%. In addition, the capture probe-modified magnetic beads (CP-MBs) showed good storage stability for at least 20 days, supporting the robustness and practical applicability of the sensing platform for routine analysis.

The applicability of the proposed genosensor was successfully demonstrated in different commercial food matrices, including soy-based beverages, rice-based beverages, and low-fat milk, achieving recovery values close to 100% with negligible matrix effects.

Overall, the developed label-free MB-based electrochemical genosensor constitutes a rapid, sensitive, cost-effective, and reliable analytical platform for Ara h2 DNA detection. The versatility of the proposed strategy, together with its simplicity and favorable analytical characteristics, makes it a promising alternative for allergen monitoring in processed foods and opens new opportunities for the development of portable and multiplexed nucleic acid biosensing platforms for food safety applications.

## Figures and Tables

**Table 1:** Oligonucleotide sequences employed in this study.

DNA Name	Oligonucleotide Sequence 5′→ 3′	Length (nt)
Target (Ara h2)	GCAGCAGTGGGAACTCCAAGGAGACAGAAGATGCCAGAGCCAGCTCGAGAGGGCGAACCTGAGGCCCTGCGAGCAACATCTCATGC	86
Capture probe (CP)	GCCCTCTCGAGCTGGCTCTGGCATCTTCTGTCTCCTTGGAGTTCCCACTGCTGC-biotin	54
Secondary probe (SP)	HS-(CH_2_)_6_-GCATGAGATGTTGCTCGCAGGGCCTCAGGTTC	32
Non-Complementary (nC)	TTCATTCAAAATAAGATCATACATACAGGTTAAAATAAACATAGGGAACCCAAATGGAAAAGGAAGGTGGCTCCTACAAATGCC	84
Non-Complementary 3-mismatches (nC-3)	GCAGCAGTGGGAACTCCAAGGAGACAGAAGATGCCAGAGCCAGCTCGAGAGGGCGAACCTGAGGCCCTGCGACCAACATCCCATCC	86

**Table 2:** Optimization of the variables Involved in the Ara h2 DNA biosensor.

Variable	Tested Range	Selected Value
CP incubation time (min)	15–60	30
CP concentration (µM)	0.5–2	1
SP concentration (µM)	0.1–2	0.5
Homogeneous hybridization time (min)	5–30	15
Heterogeneous incubation time (min)	15–60	45

**Table 3:** Precision study of the Ara h2 genosensor.

Concentration Assayed (nM)	Intra-Day Precision (RSD, %)	Inter-Day Precision (RSD, %)	Reproducibility (RSD, %)
0.05	6.4	7.2	7.9
1	5.5	7.1	7.4
20	4.8	5.5	6.2

**Table 4:** Recovery Studies of Ara h2 DNA in Commercial Food Samples.

Real Samples	DNA Ara h2 Add (nM)	DNA Ara h2 Found (nM)	Recovery (%)	RSD (%)
Soy-based beverage	0.05	0.053 ± 0.004	106	7.5
	1	1.042 ± 0.031	104.2	3.0
	10	10.060 ± 0.911	100.6	9.0
Rice-based beverage	0.05	0.052 ± 0.003	104	5.8
	1	0.987 ± 0.053	98.7	5.4
	10	9.876 ± 0.432	98.8	4.4
Low-fat cow’s milk	0.05	0.047 ± 0.004	94.0	8.5
	1	1.042 ± 0.041	104.2	3.9
	10	9.810 ± 0.812	98.1	8.4

## Data Availability

The original contributions presented in this study are included in the article. Further inquiries can be directed to the corresponding author.
